# A Versatile Brij-Linker for One-Step Preparation of Targeted Nanoparticles

**DOI:** 10.3390/pharmaceutics15051403

**Published:** 2023-05-04

**Authors:** Maria Anzengruber, Lisa Marie Nepustil, Fatlinda Kurtaj, Ammar Tahir, Katharina Skoll, Haider Sami, Michael Wirth, Franz Gabor

**Affiliations:** 1 Division of Pharmaceutical Technology and Biopharmaceutics, Faculty of Life Sciences, University of Vienna, Josef-Holaubek-Platz 2, 1090 Vienna, Austria; 2 Division of Pharmacognosy, Faculty of Life Sciences, University of Vienna, Josef-Holaubek-Platz 2, 1090 Vienna, Austria; 3 Division of Pharmaceutical Chemistry, Faculty of Life Sciences, University of Vienna, Josef-Holaubek-Platz 2, 1090 Vienna, Austria

**Keywords:** non-ionic surfactant, targeted delivery, nanoparticles, folic acid, PEGylation

## Abstract

**Background**: Most frequently the functionalization of nanoparticles is hampered by time-consuming, sometimes harsh conjugation and purification procedures causing premature drug release and/or degradation. A strategy to circumvent multi-step protocols is to synthesize building blocks with different functionalities and to use mixtures thereof for nanoparticle preparation in one step. **Methods**: BrijS20 was converted into an amine derivative via a carbamate linkage. The Brij-amine readily reacts with pre-activated carboxyl-containing ligands such as folic acid. The structures of the building blocks were confirmed by different spectroscopic methods and their utility was assessed by one-step preparation and characterization of nanoparticles applying PLGA as a matrix polymer. **Results**: Nanoparticles were about 200 nm in diameter independent of the composition. Experiments with human folate expressing single cells and monolayer revealed that the nanoparticle building block Brij mediates a “stealth” effect and the Brij-amine-folate a “targeting” effect. As compared to plain nanoparticles, the stealth effect decreased the cell interaction by 13%, but the targeting effect increased the cell interaction by 45% in the monolayer. Moreover, the targeting ligand density and thus the cell association of the nanoparticles is easily fine-tuned by selection of the initial ratio of the building blocks. **Conclusions**: This strategy might be a first step towards the one-step preparation of nanoparticles with tailored functionalities. Relying on a non-ionic surfactant is a versatile approach as it might be extended to other hydrophobic matrix polymers and promising targeting ligands from the biotech pipeline.

## 1. Introduction

Targeted therapy, by an accumulation of the drug formulation in the diseased tissue, offers the advantage of increased pharmacological activity and thus reduced dosage leading to a decreased rate of undesired adverse side effects [1,2]. One strategy to reach this aim is passive targeting by preferred non-specific interaction with the diseased tissue. In circulation, the Fahraeus effect, triggered by the central stream of red blood cells, pushes nanoparticles to the wall leading to enrichment within the smallest vessels after passing bifurcations. Another passive targeting effect of nanoparticles is the “enhanced permeability and retention” effect which was, until now, only observed in lab animals and is controversially discussed [3,4,5,6]. Additionally, in circulation nanoparticles rapidly acquire a protein corona and they are subsequently taken up by phagocytic cells, which results in rapid clearance from circulation and accumulation in the liver, spleen, and bone marrow [7,8]. As there is no active discrimination between the accumulation of nanoparticles in diseased and healthy cells, active targeting is preferable. It relies on a specific interaction between the targeting ligand on the formulation and the complementary ligand on the diseased cell. Thus the challenge is to graft a nanoparticle with different functionalities that cannot be fulfilled by one material. In this work, the main component of the nanoparticles is the poly-d,l-lactic-co-glycolic acid (PLGA), a biodegradable and biocompatible polyester that is approved for medical application by the United States Food and Drug Administration and the European Medicines Agency [9,10]. The hydrophobic nature of PLGA facilitates the loading of rather insoluble drugs and shields the cargo against harsh environmental conditions [11,12]. Drug release can be manipulated by choosing polymers with different erosion rates or external conditions such as pH or temperature [13,14]. There is a type of commercially available PLGA that offers a terminal carboxylic group for functionalization. The coupling reaction, however, is strongly limited to mild conditions to prevent particle degradation and premature release of loaded drugs. Localization close to or at the particle surface can affect the efficacy of a subsequent conjugation reaction [15]. Efficient surface modification is additionally often hampered by a dense network of an emulsifier coating the nanoparticles such as polyvinyl alcohol (PVA) inevitably necessary for the preparation of stable nanoparticle suspensions [16,17]. This steric hindrance can be seized by the insertion of a spacer molecule protruding from the particle surface. Polyethylene glycol (PEG) is one of the most commonly used hydrophilic polymers, and also lipid derivatives of PEG, are well described to introduce stealth characteristics to nanoparticles or liposomes [18,19]. At the nanoparticle surface each ethylene-oxide unit can adsorb up to three molecules of water and act as a barrier to plasma protein adsorption rendering the nanoparticle “invisible” for clearing mechanisms and prolonging the residence in circulation [20,21,22]. Brij are PEG derivatives consisting of a polyoxyethylene moiety conjugated via ether bond to a lauryl-, cetyl-, oleyl- or stearyl-hydrocarbon residue [23]. Brij nonionic surfactants are FDA-approved pharmaceutical excipients mainly used in topical formulations [24,25]. Yet there are several studies suggesting the high tolerability and suitability of Brij as an excipient in local parenteral (e.g., intratumoral) or even in intravenous formulations [26,27,28]. In structurally related non-ionic surfactants such as Myrj molecules (e.g., PEG-40-stearate) and polysorbates 20/80 (Tween20, Tween80), the hydrophobic alkyl chain and the hydrophilic PEG moiety are linked via an ester bond. Compared to the ether linkage in Brij molecules, this ester bond is higher susceptible towards oxidation and enzymatic or chemical hydrolysis. The degradation products can then themselves provoke unwanted consecutive reactions [27]. Due to the amphiphilic nature of such PEG derivatives, the PEG chain orientates itself towards the surface during the particle preparation process and the hydrophobic aliphatic tail anchors the PEG chain into the hydrophobic PLGA particle matrix [24]. To equip the nanoparticles with the ability to specifically recognize and interact with diseased cells, a targeting molecule is conjugated to the PEG moiety protruding in the surrounding prior to or post particle preparation. Folic acid is a popular tumor cell targeting molecule in the development of drug delivery vehicles, it serves as a targeting ligand since fast-proliferating cancer cells need a non-stop supply of folic acid (FA) thus expressing a considerably higher number of folate receptors than healthy cells [29,30]. Various cancers including endometrium, kidney, breast, ovary, lung and neck, colon, brain, and others are known to overexpress folate receptors [31]. In healthy human tissue, the receptor is restricted to the apical surface of the cells, however, this polarization is lost upon malignant transformation [1,5]. Furthermore, the receptor of tumor cells belongs to the alpha isoform whereas healthy cells mainly express beta-folate receptors. Nanomedicines grafted with folic acid as a targeting molecule have a higher affinity to the alpha than to the beta isoform. Additionally, healthy cells in the intestine responsible for vitamin B9 absorption preferentially interact with 5-methyltetrahydrofolate, the reduced form of the vitamin [29,32].

Surface modification of nanoparticles, however, always includes the risk of drug loss during conjugation with the targeting ligand and especially during subsequent time-consuming purification very often performed by dialysis [33,34]. Furthermore, PLGA nanoparticles are described to have a strong burst release within the first few hours in the liquid surrounding [19,35,36]. Thus, surface modification after particle preparation is a cause of drug loss and instability that should not be underestimated. A particle preparation method including the insertion of targeting molecules even during the particle preparation process could alleviate these shortcomings. Currently, mostly PEGylated phospholipids pre-conjugated with targeting ligands such as folic acid or biotin are used for this purpose [20,34,37]. These phospholipids however are rather expensive and therefore not always affordable in larger amounts for preparation of particle formulations.

In this work, a simple yet versatile and affordable method is described for using Brij-derivatives as building blocks for the preparation of functionalized nanoparticles. After synthesis and proper spectroscopic characterization, their feasibility for PLGA-nanoparticle preparation is investigated. Success and degree of functionalization were elucidated by both, chemical methods and by interaction studies of the targeted nanoparticles with single tumor cells and a cell layer of tumor target cells. Additionally, the implications of post-preparation functionalization versus one-step preparation and functionalization on the physicochemical characteristics were elucidated.

## 2. Materials and Methods

### 2.1. Materials

Poly-(lactide-co-glycolide) RG503H (PLGA) was obtained from Evonik Nutrition & Care GmbH (Essen, Germany). BodiPy 493/503 (BodiPy), HOECHST 33342 and alexa-WGA 488 (aWGA) were acquired from Invitrogen (Paisley, UK). Cell culture medium DMEM and trypsin/0.25% EDTA were bought from Gibco ThermoFisher Scientific (Waltham, MA, USA). Folic acid and 2-(4-(2-Hydroxyethyl)-1-piperazinyl)- ethansulfonic acid (HEPES) were obtained from Carl Roth GmbH (Karlsruhe, Germany). BrijS20 (MW 1152 g/mol), polyvinylalcohol (PVA; 30–70 kDa, 87–90% hydrolyzed), 1,8-diamino-3,6-dioxaoctane (DA) and all other chemicals were purchased from Sigma-Aldrich (St. Louis, MO, USA) or Merck (Darmstadt, Germany). Graphics were created with GraphPad Prism 9.0, images were created in BioRender.com, accessed on 10 January 2023 and the reaction scheme was prepared using ChemSketch 2.0.

### 2.2. Preparation of a BrijS20-1,8-Diamino-3,6-Dioxaoctane Building Block (Brij-Amine)

**Figure 1:**
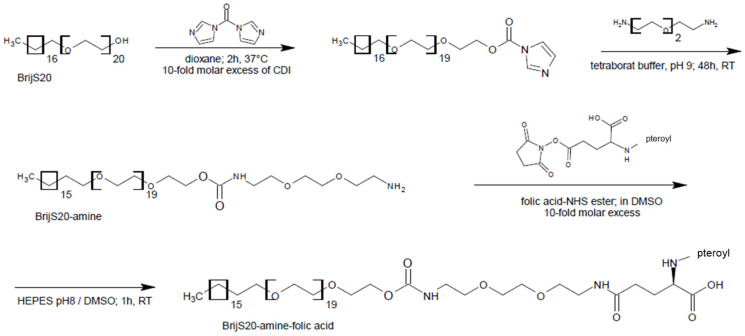
Carbonyldiimidazole-mediated activation of the hydroxyl group of BrijS20, coupling of PEG-diamine and acylation with folic acid NHS-ester.

BrijS20 (52 mM) was dissolved in dioxane at 37 °C and converted into a carbamate ester by the addition of 1,1’-carbonyldiimidazole (CDI) (0.5 M) (Figure 1). The reaction mix was stirred for two hours at 37 °C and the activated BrijS20 was then precipitated by the addition of n-hexane overnight at room temperature. After removal of the supernatant and complete solvent evaporation under reduced pressure, the remaining product was dissolved in 10 mM sodium tetraborate buffer pH 9.4. A tenfold molar excess of 1,8-diamino-3,6-dioxaoctane (DA) was added and the solution was stirred for two days. The excess reactants were removed via dialysis against purified water (MW cut off: 500 Da) and the product was lyophilized and stored at 4 °C for further use [38]. The BrijS20-amine building block now consists of the amphiphilic molecule BrijS20 and DA, providing a free amino group for further functionalization with a targeting ligand.

### 2.3. Conversion of Folic Acid to a Reactive NHS-Ester and Formation of the BrijS20-Amine-Folic Acid Conjugate

In this process, the γ-carboxylic group of folic acid is converted to an amine-reactive N-hydroxysuccinimide ester that is coupled to the BrijS20-amine building block. Briefly, folic acid (0.1 M), N-hydroxysuccinimide (NHS) (0.2 M), and dicyclohexylcarbodiimide (0.2 M) were dissolved in 100 mL anhydrous dimethyl sulfoxide (DMSO). After the addition of 2.5 mL triethylamine, the mixture was stirred at room temperature for twelve hours. The reaction mixture was then filtered to remove the by-product dicyclohexylurea. Diethyl ether was added to the filtrate to precipitate the NHS-ester of folic acid. The product was washed several times with anhydrous diethyl ether and then stored in a desiccator at room temperature until further use [39]. For conjugation, the BrijS20-amine building block was dissolved in 20 mM HEPES-buffer pH 8 and reacted with a tenfold molar excess of pre-activated folic acid NHS-ester dissolved in DMSO (Figure 1). The mixture was stirred for one hour and subsequently purified by dialysis (cut off 2000 Da) against 20 mM HEPES buffer, pH 8 [38]. Ultimately, the final product was freeze-dried and then stored at 4 °C for further use.

### 2.4. Characterization of BrijS20-Amine, Folic Acid-NHS Ester and the BrijS20-Amine Folic Acid Conjugate

The structure of BrijS20 derivatives was elucidated by ^1^H- and ^13^C-NMR-spectroscopy as well as mass spectrometry. Furthermore, UV-absorption spectra and FT-IR spectra were recorded from BrijS20, folic acid, and the final BrijS20-amine-folic acid conjugate (Supplementary Material; Figures S2 and S3). NMR analysis was performed on a Bruker instrument at 400 MHz for ^1^H-NMR and 101 MHz for ^13^C-NMR spectra. The BrijS20-amine building block and the BrijS20-amine folic acid conjugate were dissolved in deuterated water. In the case of the BrijS20-amine folic acid conjugate NaOH was added and the NMR-spectra were obtained with suppression of the water signal. The alkaline pH was necessary to dissolve sufficient amounts of the conjugate for NMR spectroscopy. Folic acid-NHS ester was analyzed in DMSO-*d_6_* due to the limited stability of the NHS-ester in water.

High-resolution mass spectrometric detection was performed using turbo ion source ESI X500 QTOF mass spectrometer (AB Sciex, Darmstadt, Germany). The BrijS20-amine conjugate was dissolved in DMSO (1 mg/mL) diluted 1:100 with methanol and 10 µL of the diluted sample was injected using a continuous flow of 95% MeCN + 0.1% formic acid at a flow rate of 350 µL/min. The following parameters were applied to achieve positive ion mode ionization: heater temperature was set to 500 °C, ion source gas 1 (30 units) was set to 30 psi, ion source gas 2 (30 units) was set to 30 psi, curtain gas (25 units) was set to 45 psi, and +5.0 KV spray voltages were used. TOF-MS scans were performed with an *m*/*z* range from 100 to 4000 using 0.05 accumulation time and −80 V decluttering potential.

### 2.5. Preparation of PLGA Nanoparticles with the BrijS20-Amine Building Block or the BrijS20-Amine-Folic Acid-Conjugate

Nanoparticles are prepared by a nanoprecipitation solvent-evaporation method [40,41]. The particles consist of a poly (lactic-co-glycolic)-acid (PLGA; 24,000–38,000 g/mol; Evonic Industries, Essen, Germany) core and the BrijS20-amine-(folic-acid)-conjugate, which is anchored into the PLGA matrix by its carbon chain during the particle preparation process. The hydrophilic polyethylene glycol chain protruding from the particle directs the free amino group or the already conjugated targeting molecule outwards. Briefly, 50 mg PLGA, BodiPy (25 µg) as a fluorescence marker to visualize the particles in cell-binding and internalization assays, and varying amounts of the previously prepared BrijS20-amine or BrijS20-amine-FA-conjugate, were dissolved in 3 mL of a mixture consisting of ethyl acetate and acetone in a 1:4 ratio. The solution was aspired into a syringe (cannula 0.8 × 40 mm) and injected into 30 mL 10 mM HEPES-buffer pH 6.5 containing 0.5% polyvinyl alcohol (PVA) as a stabilizer and emulsifier. The mixture was treated with an ultrasonic homogenizer (Bandelin, UW 70) for three minutes at an intensity of 40% during constant agitation and cooling.

In the case of the BrijS20-amine-FA-conjugate the organic phase was treated with ultrasound for 30 s immediately before injection to ensure complete homogenization of the hardly soluble conjugate. All further preparation steps remained the same. After the formation of a stable suspension, the reaction mix was transferred to a 1.5 times larger volume of the same aqueous phase as described above and stirred at a constant speed (300 rpm) and under continuous airflow for one hour to evaporate the organic solvent and to harden the nanoparticles. Subsequently, the particles were purified by consecutive centrifugation steps at 4 °C at 119× *g*, 11,872× *g*, 20,064× *g*, 30,392× *g,* and 42,858× *g* for 30 min each. The final supernatant was discarded. Also, the first pellet was discarded to remove larger aggregates and the remaining particle pellets were suspended in 0.5% aqueous PVA solution, pooled, freeze-dried, and stored at 4 °C for further use.

Adhering to the conventional post-preparation functionalization protocol, nanoparticles prepared with the BrijS20-amine building block were subsequently conjugated with NHS-folate as a targeting molecule. To this end, PLGA-BrijS20-amine particles were dispersed in 20 mM HEPES-buffer pH 8, and a tenfold molar excess of preformed folic acid NHS-ester dissolved in DMSO was added to the particle suspension. The amount of folic acid-NHS ester was calculated with reference to the amino groups on the particle surface. After one hour at room temperature, the excessive folic acid was removed by centrifugation (20,817× *g*, 20 °C, 15 min) from the particle suspension. The final folate-functionalized nanoparticle suspension prepared by a post-preparation functionalization protocol was suspended in 0.5% PVA solution, lyophilized, and further stored at 4 °C. All particle preparations investigated contain a total of 0.5% PVA as cryoprotectant and for stabilization of the particle suspension.

### 2.6. Characterization of Particle Size and Zeta Potential

Particle size distribution and polydispersity index (PDI) of all nanoparticle suspensions before and after lyophilization were determined in triplicates by dynamic light scattering at a backscattering angle of 173° using a Zetasizer Nano ZS (Malvern Panalytical, Malvern, UK). Furthermore, the zeta potential of all particle suspensions was determined also using a Zetasizer Nano ZS, (Malvern Panalytical, Malvern, UK). An automatic selection for the number of runs was chosen. Lyophilized nanoparticles were suspended in the same volume of purified water as before lyophilization. The particle concentration of all suspensions was adjusted by dilution with 0.5% PVA solution to 0.3 mg/mL nanoparticles to ensure the comparability of the results. Additionally, the particle size of PLGA/BrijS20 nanoparticles and PLGA/BrijS20-amine-FA-conjugate nanoparticles was determined by nanoparticle tracking analysis (NTA) using a NanoSight NS500 (Malvern Panalytical, Malvern, UK) (Supplementary Material).

### 2.7. Quantification of Primary Amine Groups and Particle-Associated Folic Acid

Upon incorporation of the BrijS20-conjugates described above into the particle matrix, either free amine groups or folic acid are present on the surface of the PLGA particles. The number of BrijS20-amine conjugates successfully integrated into the particle-matrix or the decrease in the number of primary amine groups due to folate coupling was determined with an amine-reactive fluorescent reagent. The assay was performed according to an optimized protocol by Thermo Fisher Scientific, Waltham, MA, USA, described in the instructions for Fluoraldehyde Reagent Solution. The reagent to be prepared in advance consisted of 7 mM o-phthalaldehyde and 6 mM mercaptoethanol in 20 mM HEPES buffer pH 10. The dry particles were dispersed in purified water and mixed with the previously prepared amino-reactive reagent at a 4 + 1 ratio (*v*/*v*) and transferred into a 96-well plate for fluorescence measurements. After five minutes, the fluorescence intensity was read at 340/455 nm (excitation/emission) using a microplate reader. All samples were analyzed after equal time intervals to attain the highest accuracy and reproducibility. A 0.5% PVA solution was used as a negative control and subtracted from the determined values. The test was calibrated with DA.

The amount of folic acid conjugated to the particle surface was quantified by measuring the absorption of the targeting molecule at 365 nm in a microplate reader (Tecan InfinitePro, Grödig, Austria). The dried particles were suspended in purified water and dissolved by adding a 4 M sodium hydroxide solution. This minimizes the unspecific absorption of the particle suspension and makes the quantification of folic acid more accurate. Nanoparticles prepared without the fluorescent dye BodiPy were used for quantification of primary amines as well as folic acid to not interfere with the results of the measurements.

### 2.8. Binding and Internalization of BodiPy-Labelled PLGA Nanoparticles

Potential internalization and binding properties of the particle suspensions were investigated using both KB single cells and monolayer. The KB cell line is a human epithelial carcinoma cell line expressing folate receptors. The cells were obtained from ATCC and cultivated in DMEM cell culture medium fortified with 10% fetal bovine serum at 37 °C in a humidified 5% CO_2_/95% air atmosphere. According to the supplier´s instructions cells were sub-cultivated using an aqueous solution containing 0.25% trypsin and 0.3% EDTA. For binding studies, the assays were performed at 4 °C to downregulate active transport into the cell, whereas internalization is upregulated at 37 °C.

For monolayer experiments, KB cells were cultivated in 96-well plates. Prior to particle incubation, the cell culture medium of the confluent monolayer was removed by washing twice with 20 mM isotone HEPES pH 7.4. Then 80 µL of the previously prepared particle suspension (0.4 mg/mL) in isotone HEPES pH 7.4 were added to each well and incubated at 37 °C for two hours. Excess particles were then removed by washing with buffer and the amount of cell-associated BodiPy labeled particles was assessed at 485/525 nm (excitation/emission) in a microplate reader.

For cell suspension experiments, 50 µL of particle suspension in isotone HEPES 7.4 were incubated with the same volume of the cell suspension (3 × 10^6^ cells/mL) for two hours at 4 °C or 37 °C. Excess particles were removed by centrifugation at 106× *g*, 4 °C for five minutes and the supernatant was discarded. The bound and internalized particle fractions were analyzed by flow cytometry. To ensure comparability between the particle suspensions applied, the fluorescence intensity was adjusted to the same level by dilution resulting in an average particle concentration of 0.4 mg/mL.

### 2.9. Microscopic Analysis of the Particle-Cell Interaction

The potential internalization properties of nanoparticles were further examined at 37 °C and visualized by fluorescence microscopy. KB cells were cultivated on a coverslip in Flexiperm (Fa. Sarstedt, Nümbrecht, Germany). After washing the confluent cell monolayer in each well with isotone HEPES-buffer pH 7.4, the cells were incubated for two hours with 100 µL DMEM with or without nanoparticles. Subsequently, the cells were washed with isotone HEPES and fixed by incubation with 100 µL 2% paraformaldehyde for ten minutes at 4 °C. After inactivation of paraformaldehyde by the addition of ammonium chloride to reach a final concentration of 50 mM for further ten minutes at room temperature, the cells were washed with isotone HEPES buffer pH 7.4. For co-localization, the cell nucleus was stained with the DNA-specific dye HOECHST 33342 (blue; final concentration 36 µg/mL) and the cell membrane was labeled with alexa-Fluor 488 WGA at 4 °C for 30 min (red; final concentration 24 µg/mL). The cell layers were examined using a fluorescence microscope (Zeiss Epifluorescence Axio Observer.Z1 microscopy system, Carl Zeiss, Oberkochen, Germany).

### 2.10. Statistical Analysis

Data are expressed as the mean of at least three independent experiments ± standard deviation. Statistical analysis of the results was performed using GraphPad Prism 9. For two-group comparison statistical significance was determined using a two-tailed unpaired *t*-test. Multiple group comparison was analyzed by one-way ANOVA followed by Tukey-Kramer multiple comparison posthoc analysis. A difference with a *p*-value below 0.05 was considered statistically significant.

## 3. Results and Discussion

### 3.1. Synthesis and Spectroscopic Characterization of Brij-Amine and BrijS20-Amine Folic Acid Conjugate

**Figure 2:**
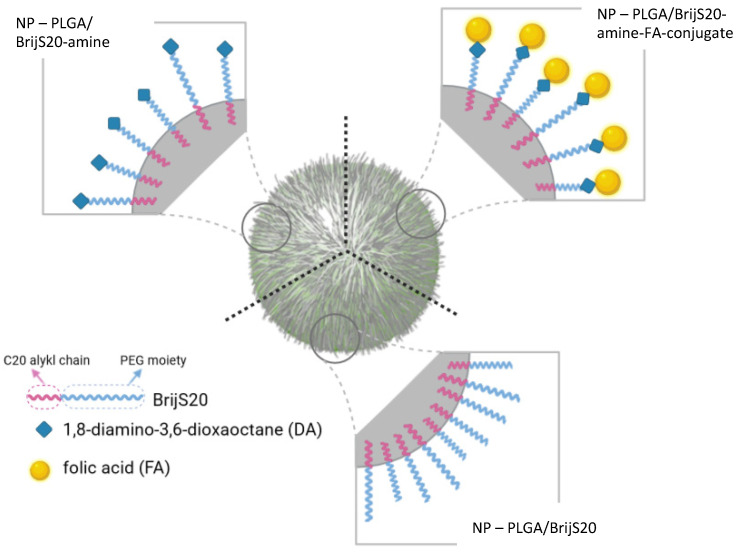
Suggested particle morphology: The hydrophobic stearyl moiety of BrijS20 is inserted into the PLGA particle matrix; the hydrophilic PEG moiety covers the particle surface. The hydroxyl groups can be modified with an amine group (blue square) as an anchor for further molecules and further with folic acid (yellow dot) as a targeting ligand.

The rationale for selecting Brij as a backbone for the building block was, on one hand, the possible interaction of the stearyl ether-moiety with the lipophilic PLGA molecules to allow for the preparation of particles. On the other hand, the hydrophilic PEG moiety is expected to protrude into the hydrophilic surrounding and act as an entropic barrier against protein adsorption [24]. Furthermore, since the Brij molecule offers only one easily accessible hydroxyl group for further functionalization, unidirectional synthesis is perfectly obvious. The Brij type S20 was chosen because of its favorable ratio between hydrophilic and lipophilic proportions. The rather long C20 alkyl chain is stably inserted into the PLGA matrix whereas the PEG moiety sticks out into the surrounding and thereby increases the hydrophilicity of the particle surface. In the case of unmodified Brij with a terminal hydroxyl group, this conformation can potentially reduce unspecific adhesion of proteins and mediate biorecognitive interactions upon modification of the hydroxyl group with a targeting ligand (Figure 2). Also, the PEG chain functions as a spacer between the particle surface and the respective targeting ligand. This provides more flexibility during a possible interaction with the target site [42].

To convert the hydroxyl group of Brij into a conjugation-friendly amine group a short-chained PEG-diamine was attached via a carbamate linkage mediated by carbonyldiimidazole (Figure 1). As a side effect, this reaction also elongated the PEG-moiety of the reactive amphiphilic building block and probably improved the performance of the “stealth building-block”. To extend the utility of the “stealth building block” towards targeting characteristics, the low molecular weight targeting ligand folic acid was conjugated in a two-step procedure. First, folic acid was converted in the presence of carbodiimide into a reactive N-hydroxy-succinimide ester. Second, the active ester acylates the free amine group of Brij-amine creating a stable amide linkage. Thus, the targeting block comprises three functionalities: a hydrophobic domain for association with PLGA, a hydrophilic domain for limited opsonization and a targeting domain for site-specific interaction.

^1^H- and ^13^C-NMR spectra confirmed the formation of both intermediate building blocks as well as the final BrijS20-amine-folic acid conjugate. In the NMR spectra of folic acid NHS-ester characteristic signals from the pteroyl partial structure and the glutamic acid structure of folic acid as well as a pronounced signal from the NHS-ester at δ 2.57 ppm (^1^H-NMR) and δ 25.18 ppm (^13^C-NMR) were observed. Upon analysis of the BrijS20-amine building block, strong signals from the PEG moiety (^1^H-NMR: δ 3.53–3.88 ppm and ^13^C-NMR: δ 69.72 ppm), as well as the alkyl structure (^1^H-NMR: δ 1.58–0.91 ppm and ^13^C-NMR: δ 26.23–13.94 ppm), were detected. Additionally, a peak originating from the CH_2_ group next to the ester moiety and signals from the CH_2_ groups neighboring the primary and secondary amine was observed. The final BrijS20-amine-folic acid conjugate contained signals from the PEG- and alkyl structure characteristic for the Brij-component as well as distinctive signals from the pteroyl- and glutamic acid structures of folic acid, therefore indicating the successful conjugation. Yet, despite a lengthy purification procedure, signals from residual HEPES buffer were detected in the NMR spectra of the final BrijS20-amine folic acid conjugate. Also, triethylamine was not entirely removed from the folic acid NHS-ester intermediate product. Therefore, to improve accuracy, further experiments are focusing on increasing the efficiency of the purification process. These impurities, however, did not seem to interfere with the reproducible formation of folic acid-targeted nanoparticles.

In the mass spectrum of BrijS20-amine-folic acid a mass peak from the final compound could be found at 1749 Da. Interestingly, an additive ion series of 44 Da was observed which corresponds with the molar mass of a PEG subunit. According to PEG polymer adduct formation however, the [M + H] ion should be 3 Da higher than observed in the ion series. The dominant “3 Da less” ion species could be explained as abstracted hydrogen cations since there are two amides and one carbamate bond in the final product. This was previously reported in studies including comparable compound chemistries [43,44]. In accordance with the results from NMR spectroscopy, residual HEPES buffer as well as non-reacted folic acid was detected by mass spectrometry. Also, a certain inhomogeneity in the degree of polymerization regarding the PEG units in the parent Brij molecule became apparent. All in all, the chemical structure of the Brij-amine-folic acid conjugate proven by NMR spectroscopy was confirmed by mass spectrometric data. The detailed peak assignment as well as the NMR spectra (Figures S4–S10) and MS spectrum (Figure S11 and Table S1) can be found in the Supporting Material.

### 3.2. Preparation and Characterization of Nanoparticles

**Figure 3:**
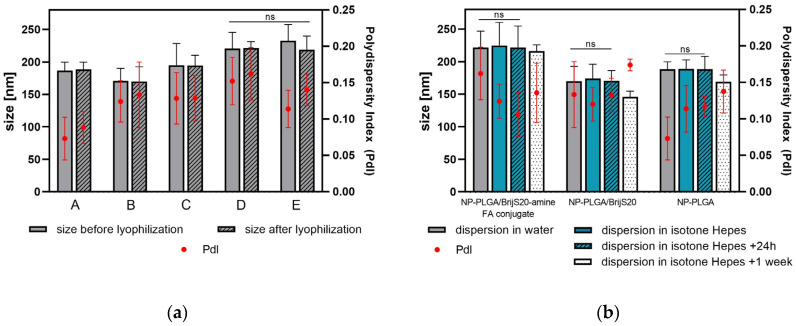
(**a**) Particle size and PdI before and after the lyophilisation process of all particle suspensions prepared; A: plain PLGA-NPs; B: PLGA/BrijS20-NPs; C: PLGA/BrijS20-amine-NPs, D: PLGA/BrijS20-amine-folic acid conjugate-nanoparticles (one-step particle preparation); E: PLGA/BrijS20-amine + folic acid—NPs (post-preparation particle functionalization); (**b**) particle size and PdI after dispersion in water or isotone HEPES buffer pH 7.4. The size and size distribution of particle suspensions in HEPES—buffer stored at 4 °C were determined after 24 h and after one week.

**Figure 4:**
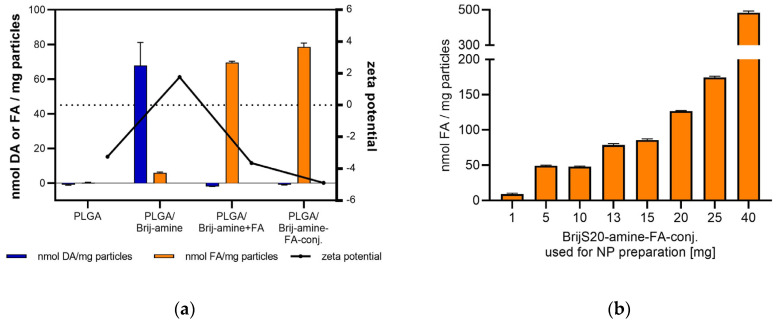
(**a**) Zeta potential and amine or folate density of PLGA nanoparticles prepared in one step (PLGA, PLGA/Brij-amine and PLGA/Brij-amine-FA) or stepwise (PLGA/Brij-amine + FA). Poly-d,l-lactide-co-glycolide (PLGA), BrijS20 (Brij), 1,8-diamino-3,6-dioxa-octane (DA), folic acid (FA). Nanoparticle suspensions were prepared with 13 mg Brij-amine or Brij-amine-FA. (**b**) The density of folic acid on the particle surface correlates with the amount of BrijS20-amine-folate used for nanoparticle preparation.

By combining nanoprecipitation with solvent evaporation nanoparticles were prepared from PLGA as a matrix polymer. The non-ionic surfactant Brij-amine was readily soluble in the organic layer, but the Brij-amine folate possessing features of an ionic surfactant was hardly soluble. It was readily soluble in a slightly alkaline medium that vice versa degraded the polyester nanoparticles. The compromise was to generate the finest dispersion by ultrasonication and to use HEPES-buffer pH 6.5 for precipitation. Applying this one-step procedure, nanoparticles were prepared from PLGA, PLGA/Brij-amine, and PLGA/Brij-amine-FA (PLGA/Brij-amine-FA-conj. in Figure 3 and Figure 4). For comparison, PLGA-Brij-amine nanoparticles were grafted with the same ligand post-particle preparation (referred to as PLGA/Brij-amine + FA).

All nanoparticles were around 200 nm in diameter with a PDI below 0.2 indicative of monodisperse suspensions (Figure 3). Nanoparticles prepared with BrijS20, BrijS20-amine, or PLGA alone tended to be smaller as compared to nanoparticles with folic acid as a targeting moiety. The difference in the size of about 20 nm was also confirmed by NTA experiments (Supplementary Material, Figure S3). With regard to particle size, no difference between nanoparticles modified with folic acid according to a one-step protocol or a post-preparation functionalization protocol was observed (*p* > 0.5). The lyophilisation process did not alter particle size or size distribution. The dried particles could be stored over several months without any sign of degradation or reduced stability confirming the adequate choice of the PVA concentration as a cryoprotectant. The dried particles were easily re-dispersible in purified water or isotone HEPES buffer. Particle sizes observed in the HEPES buffer did not vary significantly from those measured in purified water. Even 24 h after resuspension no signs of agglomeration or significant change in size or PdI were observed. After one week of storage in suspension the particle size decreased. This slight decrease in particle size was more pronounced for PLGA/Brij nanoparticles than for plain PLGA particles or folic acid-modified particles prepared according to the one-step preparation protocol. This might be due to the emulsifying capacity of the Brij molecule. Although the particle preparations exhibited good stability in suspension, considering future incorporation of an API and washout of the drug that might occur during storage in suspension, the nanoparticles were stored dry at 4 °C and were suspended in isotone HEPES immediately before cell experiments. Although the impact of the Brij-component on drug loading and drug release was not yet investigated, we assume that the release will be accelerated due hydrophilization of the particle surface [14]. In terms of drug loading, we speculate that the emulsifying properties of Brij might reduce the initial drug loading by increasing the solubility of lipophilic drugs in the aqueous surrounding. This effect is certainly influenced by the hydrophilicity inherent to the drug as well as the concentration of Brij during the particle preparation process. Investigation of drug loading and release will be part of consecutive experiments.

When analyzing the zeta potential at least in the case of PLGA-NPs made from polyesters with free carboxylic end groups a high negative zeta potential would have been expected, but low values were obtained. This indicates high shielding of the surface charges by PVA, which was inevitably necessary for the stabilization and cryoprotection of the particle suspensions [45,46]. The lowering of the surface potential caused by the neutral polymer PVA coating the particle surface did not impede particle stability. As described by Robin et al. stabilization of PVA-containing particle suspensions is facilitated by steric hindrance rather than electrostatic repulsion [47]. Yet, although the zeta potential ranged around the zero point, the different particle-surface modifications caused clear changes in the zeta potential (Figure 4a). PLGA particles with free carboxylic groups had a negative zeta potential of around −3 mV [15]. Similar zeta potential values around −3 mV were observed for PLGA particles prepared with unmodified BrijS20. Incorporation of the BrijS20-amine conjugate into the particle matrix shifted the zeta potential to a slightly positive range. This shift reflects the high number of amino groups present at the hydrodynamic surface of the particles [16]. After neutralizing the basicity of the amino groups by coupling folic acid, the zeta potential dropped back to negative values. Since folic acid introduces an additional carboxylic group at the particle surface the zeta potential was lower than that of unmodified PLGA particles. A similar zeta potential of around −5 mV was observed for particles prepared with the preformed BrijS20-amine-FA conjugate.

The amount of BrijS20-amine attached to the particle matrix was measured to be as high as 70 nmol amine groups per milligram nanoparticles. This corresponds to a molar ratio of PLGA to Brij-amine of around 1:2 to 1:3 due to the high variance in molecular weight (24–38 kDa) of the PLGA component. Conjugation of activated folic acid in the next steps caused the amino groups to disappear below the detection limit. Concurrently, a comparable molar amount of about 69.5 nmol folic acid was bound to the particle surface. Thus, all the amino groups present at the surface were accessible for functionalization. Unfortunately, the yield of amine-modified nanoparticles was reduced indicating the dissolution of the nanoparticles. Most probably this was caused by particle-inherent amine-promoted hydrolysis of the PLGA polyester and the emulsifying character of the Brij molecule. This effect was only observed upon stepwise preparation of the nanoparticles. Upon one-step preparation, however, this premature dissolution tendency was not observed. Interestingly, 78.6 nmol folic acid was grafted at the surface of the nanoparticles, which is still in the same range as the stepwise preparation.

To avoid the critical influence of the free amine-group and to obtain an idea of the utility of the one-step preparation method with tailored building blocks, the adjustability of the targeting ligand density was examined. To this end the weight ratio of PLGA:BrijS20-amine-FA used for particle preparation was constantly increased from 50:1 to 50:40. Determination of the folate content of the nanoparticles revealed a clear correlation between the two parameters. The number of folic acid molecules on the particle surface increased from 9 nmol/mg particles to 480 nmol/mg particles in a concentration-dependent manner covering a wide range of adaptability (Figure 4b). Keeping in mind that even the one-step preparation method applied involves manufacturing and purification steps that can potentially influence the outcome, the calculated correlation coefficient R^2^ of 0.995 over the whole concentration range when applying an exponential fit, indicates a high reproducibility and clearly shows that the targeting ligand density at the nanoparticle surface can be fine-tuned by the particle preparation process (Supplementary Material).

### 3.3. Interaction of Nanoparticles with KB Cells

To obtain an idea of the interaction of the folate-modified particles with cells, in-vitro experiments with folate receptor-expressing KB cell monolayers as well as single cell suspensions were conducted. To allow for fluorimetric quantification of the interaction, nanoparticles were loaded with the label BodiPy within the first step of nanoparticle preparation.

**Figure 5:**
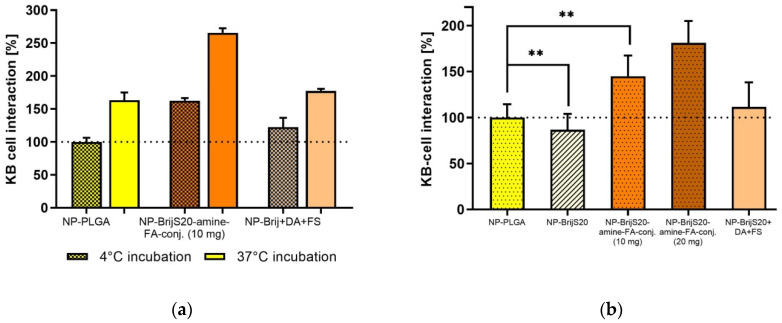
Cell binding and internalization of targeted and non-targeted nanoparticles in cell suspension assays (**a**) after incubation for two hours at 4 °C (hatched area) and 37 °C (plain area) and monolayer experiments (**b**) (KB cells). Cell binding was examined at 4 °C whereas uptake was analyzed at 37 °C. Results of monolayer and cell suspension assays using flow cytometry (FACS) are depicted in relation to the cell-associated fluorescence intensity of unmodified PLGA particles (at 4 °C). ** *p* < 0.01.

In single-cell experiments, the amount of cell-associated nanoparticles was quantified by flow cytometry. Performing the assay at 4 °C provokes a lack of energy and suppresses active transport processes so that predominantly binding of the nanoparticles to the cell membrane was observed (Figure 5a). Due to hydrophobic interactions cell binding of blank PLGA-nanoparticles occurred at 4 °C. At 37 °C additional adsorptive endocytosis contributes to cell interaction and increased the cell-association. In the case of folate-functionalized nanoparticles, the cell-binding at 4 °C as well as the additional intracellular uptake at 37 °C considerably exceeded that of the blank particles up to 1.6-fold. The one-step preparation procedure yielded folate nanoparticles with higher cell-binding and internalization rates although the folate content of both nanoparticle types was comparable. Although this is a repeatable result, up to now there is no satisfying explanation and this discrepancy is and will be the subject of further investigations.

The monolayer experiments were conducted at 37 °C and the bound fraction of labeled nanoparticles was determined by fluorescence reading (Figure 5b). Interestingly, the cell interaction of PLGA/Brij nanoparticles is significantly lower than that of blank PLGA nanoparticles. Thus, the PEG structures of the PLGA/Brij nanoparticles reduced the non-specific, hydrophobic interaction and are strong evidence for the stealth properties of the nanoparticles mediated solely by the BrijS20-content. The presence of folate as a targeting ligand further increased cell-binding and uptake by 45%. When the amount of Brij-amine-folate was doubled, the amount of cell-associated nanoparticles increased by 81% as compared to blank PLGA nanoparticles. This confirmed a targeting ligand-density-dependent interaction. Again, even in monolayer experiments, the cell association of post-preparation functionalized nanoparticles was 35% lower than the cell interaction of one-step prepared ones.

All in all, the in-vitro assays in both models, single cells and a monolayer of folate receptor-expressing cells, confirmed that the integrity and functionality of the targeting ligand within the nanoparticles were retained. The observation that increased targeting ligand density leads to stronger interaction might be useful for experiments under hydrodynamic flow conditions. Moreover, the translation of the protein-repellent characteristics of the PEG-moiety of BrijS20 to the cell association of nanoparticles was underlined which renders the nanoparticles “targeted” and “stealth”. In contrast to our experiments, the stealth effect of PEG is mostly described in the literature for PEG molecules with a molecular weight over 2000 [48,49]. Although controversially discussed in the literature, we hypothesize that the combination of the PVA present in the particle sample (0.5% *w*/*v*) together with the Brij-induced PEG coating might be the reason for the reduced particle cell interaction observed in in-vitro experiments [22,50,51].

### 3.4. Microscopic Analysis of Particle Internalization in KB Cells

**Figure 6:**
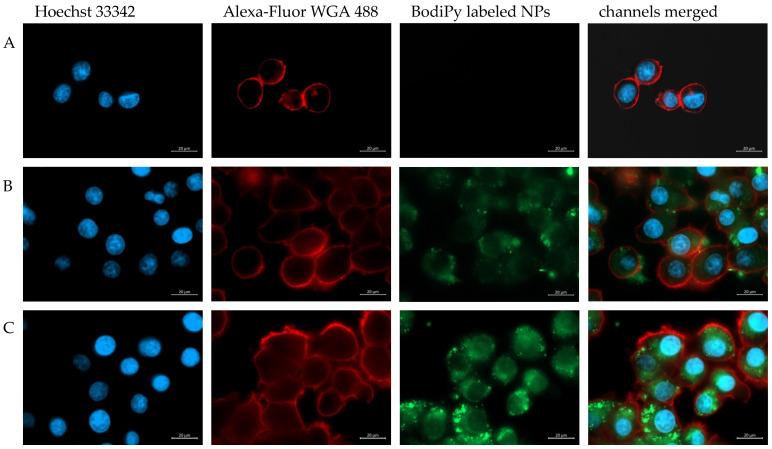
Visualization of the cellular uptake of BODIPY labeled nanoparticles (green); KB-cells were incubated with DMEM (**A**) or the respective nanoparticle formulation ((**B**): PLGA-nanoparticles; (**C**): PLGA/Brij-amine-FA-nanoparticles) for two hours at 37 °C and after fixation in paraformaldehyde with the nucleus stain HOECHST 33342 (blue) and the membrane stain alexa-fluor 488 WGA (red). Scale bar indicates 20 µm.

To visualize a possible cell internalization of modified particles, BodiPy-labelled PLGA particles functionalized with folic acid were prepared and cytoinvasion was observed on a KB cell layer. Fluorescence microscopic images revealed that after an incubation period of two hours at 37 °C as described in Section 2.6 nanoparticle suspensions showed a certain cytoinvasive potential. However, a markedly increased cellular uptake of PLGA/BrijS20-amine-folate nanoparticles (Figure 6C) compared to non-modified PLGA nanoparticles (Figure 6B) can be observed. These results are in good accordance with the particle-cell interaction behavior observed in monolayer and single-cell experiments.

## 4. Conclusions

In this work, BrijS20 is proposed as a basic structure for different building blocks. The hydrophobic moiety of the non-ionic surfactant is anchored in the PLGA-polyester and the polyethylene-glycol moiety might be antennary exposed at the surface. According to the interaction with KB-cells, the surfactant as a building block of nanoparticles reduces cell association by 13% as compared to nanoparticles without surfactant. This stealth effect possibly reduces non-specific particle cell adhesion and is expected to mitigate unwanted side effects of drug therapy. To mediate versatility, the terminal hydroxyl group was converted to a readily reactive amine by extension with a diamine-ethylene-glycol elongating the antenna and probably enhancing the stealth effect. Moreover, this free amine group is easily acylated by activated carboxylic compounds of cell-specific ligands. The coupling of pre-activated folic acid as a targeting ligand gave rise to a targeting building block. The retained functionality of this building block was underlined by at least 1.6-fold higher binding of nanoparticles to folate-receptor expressing KB-cells as compared to blank nanoparticles. Although folate is a highly stable targeting ligand, the formation of a targeting building block is expected to be extended to ligands from the biotech pipeline with delicate molecular structures. This is currently under investigation and will be the subject of further manuscripts. Additionally, the targeting ligand density is easily fine-tuned by a selection of the weight ratio between matrix polymer and building block. Also, the preparation of Brij-conjugates is one-directional due to the use of the single free hydroxyl group as an anchor for further functionalization. The here-developed Brij-conjugates as well as the conjugation process have the potential to at least partly replace highly expensive PEGylated phospholipids not only in polymeric particle formulations but also in liposomal formulations. The main advantage of the preparation of nanoparticles from building blocks with different functionalities, however, is the one-step preparation. This prevents many time-consuming single steps, the associated loss of drug content, and the use of harsh reaction conditions. According to the building blocks available, nanoparticles with tailored functionalities are expected to be prepared by simply adjusting the ratio between matrix polymer, stealth moiety, and targeting moiety. Thus the presented BrijS20 conjugates are versatile and affordable tools for the preparation of targeted nanoparticles in one manufacturing step.

## Data Availability

Data will be made available upon reasonable request.

## Supplementary Materials

The following supporting information can be downloaded at: https://www.mdpi.com/article/10.3390/pharmaceutics15051403/s1, Figure S1: Cytotoxicity assay; Figure S2: calculated exponential correlation; Figure S3: Particle size and size distribution determined by NTA; Figure S4: UV-absorption spectra; Figure S5: FT-IR spectra; Figure S6: ^1^H-NMR of BrijS20-amine in D_2_O; Figure S7: ^13^C-NMR of BrijS20-amine in D_2_O; Figure S8: ^1^H-NMR of BrijS20-amine in D_2_O; Figure S9: ^13^C-NMR of folic acid NHS-ester in DMSO-*d_6_*; Figure S10: ^1^H-NMR of BrijS20-amine folic acid in D_2_O/NaOH; Figure S11: ^13^C-NMR-DEPT of BrijS20-amine folic acid in D_2_O/NaOH; Figure S12: ^13^C-NMR of BrijS20-amine folic acid in D_2_O/NaOH; Figure S13: mass spectrum of BrijS20-amine-folic acid conjugate. The m/z region of the final product [3] is enlarged. Table S1: exact mass and [m + H] of characteristic peaks in the mass spectrum of the BrijS20-amine-folic acid conjugate.
