# Supplementary material for: A Versatile Brij-Linker for One-Step Preparation of Targeted Nanoparticles
Source: Pharmaceutics. 2023 May 4;15(5):1403. doi: 10.3390/pharmaceutics15051403 (PMC10222160; doi:10.3390/pharmaceutics15051403)

*Supplementary material to the manuscript*

# A versatile Brij-Linker for one-step preparation of targeted nanoparticles

*Maria Anzengruber[1*], Lisa Marie Nepustil[1], Fatlinda Kurtaj[1], Ammar Tahir[2], Katharina Skoll[1], Haider Sami[3] Michael Wirth[1], Franz Gabor[1]*

[1] University of Vienna, Faculty of Life Sciences, Division of Pharmaceutical Technology and Biopharmaceutics, Josef-Holaubek-Platz 2, 1090 Vienna, Austria

[2] University of Vienna, Faculty of Life Sciences, Division of Pharmacognosy, Josef-Holaubek-Platz 2, 1090 Vienna, Austria

[3] University of Vienna, Faculty of Life Sciences, Division of Pharmaceutical Chemistry, Josef-Holaubek-Platz 2, 1090 Vienna, Austria

***Cytotoxicity assay – Methods and Results***

The potential cytotoxic activity of the particle formulations on KB cells as well as 5637 human urothelial cancer cells was assessed by a commercially available XTT assay (EZ4U-XTT assay kit, Biomedica Medizinprodukte GmbH & Co KG, Vienna, Austria). The second cell line was added in order to elucidate possible cell type specific differences in cytotoxicity of the particle formulations. The assay was conducted according to supplier's instructions. In brief, cells were seeded in a 96-well plate at a density of 10,000 cells per well and cultivated for 12 h in DMEM medium without phenol red. The particle suspensions were diluted to obtain a concentration of 1 mg/ml and 0.5 mg/ml nanoparticles. According to calculations this corresponds to about 80 µg/ml and 40 µg/ml BrijS20. Cells were incubated with the particle suspensions until the negative control (untreated cells) reached confluency. Then the supernatant was replaced by fresh medium and 20 µl XTT-reagent were added to each well. Monolayers were further incubated for two hours at 37°C and the absorbance was read in a microplate-reader at 450 nm using 620 nm as a reference.

Cell viability was compared after incubation with plain PLGA-NPs, PLGA/BrijS20-NPs and folic acid targeted nanoparticles prepared in one step (PLGA/BrijS20-amine-FA conjugate-NPs). Overall results showed acceptable tolerability of all particle formulations. Further no significant concentration dependent cytotoxicity was observed in these experiments. Nevertheless incubation with particles incorporating a BrijS20 moiety showed an around 10% reduced cell viability compared to after incubation with plain PLGA-NPs. This was observed for both cell lines. Generally 5637 cells exhibited higher cell viability compared to KB cells. This is in accordance with the observation that 5637 cells form a more stable monolayer and reach a higher level of confluency. Interestingly, PLGA/BrijS20 nanoparticles appear to be slightly more toxic in both cell lines compared to the folic acid targeted particles (Figure S1). Yet this difference was not statistically significant. Nevertheless it underpins the slight differences observed for PLGA/BrijS20 nanoparticles in other experimental conditions such as particle stability and cell binding.

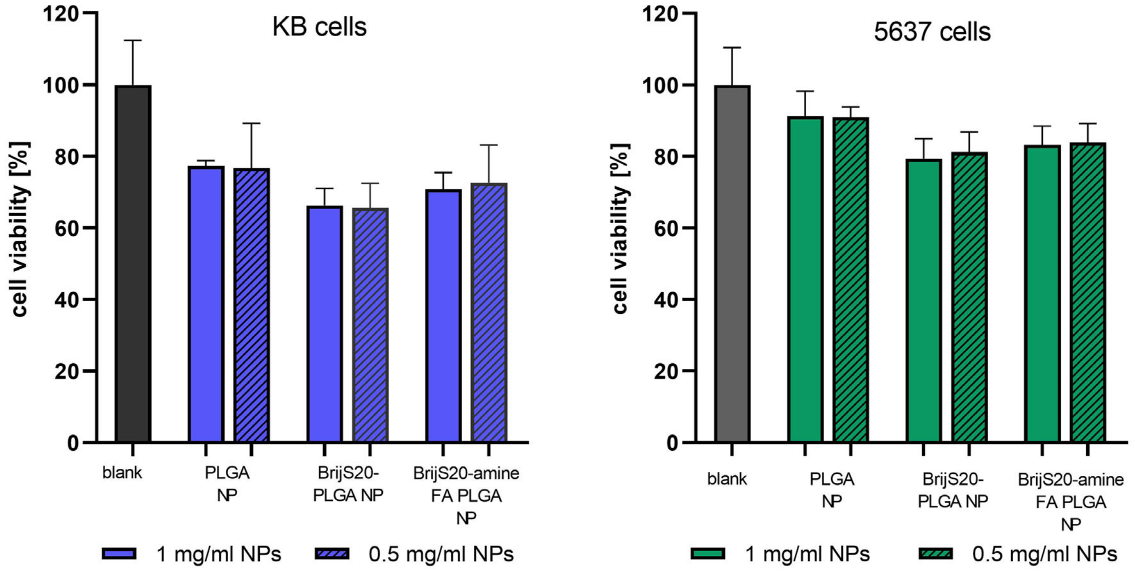


**Figure S1:** Cytotoxic potential of nanoparticle formulations on KB cell layers and 5637 cell layers. Cell viability is compared to untreated cells (blank).

***Customizing the amount of folic acid on the particle surface***

The correlation of BrijS20-amine-FA-conjugate used for particle preparation and the actual amount of targeting ligand was investigated over a concentration range of 1 – 40 mg Brij-conjugate used. The number of folic acid molecules on the particle surface increased from 9 nmol/mg particles to 480 nmol/mg particles in a concentration dependent manner covering a wide range of adaptability (Figure 4b). Keeping in mind that even the one-step preparation method applied involves manufacturing and purification steps that can potentially influence the outcome, the calculated correlation coefficient $R^2$ of 0.995 over the whole concentration range when applying an exponential fit, indicate for high reproducibility and clearly show that the targeter density at the nanoparticle surface can be fine-tuned by the particle preparation process. The calculated exponential correlation is given in Figure S2 below.

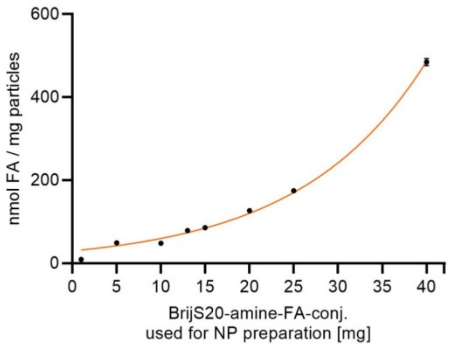


**Figure S2:** Calculated exponential correlation in the concentration range of 1-40 mg BrijS20-amine-folate conjugate used for particle preparation. The correlation coefficient $R^2$ is 0.995.

**Characterization of size using nanoparticle tracking analysis (NTA)**

In addition to size characterization by dynamic light scattering (DLS) using the ZetaSizer Nano ZS (Malvern Panalytical, Malvern, UK) PLGA/BrijS20 nanoparticles and PKGA/BrijS20-amine-FA-conjugate nanoparticles

were analysed by NTA using a NanoSight NS500 (Malvern Panalytical, Malvern, UK). Prior to size measurements, particles were dispersed in 0.5% PVA solution and further diluted in 0.5% PVA at a concentration range of $10^8$ to $10^9$ particles per ml to finally achieve 20-100 particles in the field of view during NTA analysis. NTA measurements were performed at 25°C and with 488 nm laser. Five measurements, each with a duration of one minute, were acquired and data was analysed with NTA 3.1 software (Malvern Panalytical, Malvern, UK).

Results showed similar sizes for both particle preparations analysed. The mean size of PLGA/BrijS20 nanoparticles was calculated to be 197 nm whereas the mean size of PLGA/BrijS20-amine-FA nanoparticles was around 18 nm higher. This corresponds well with the results obtained from the DLS measurements (Figure S3).

**PLGA/BrijS20-NPs**

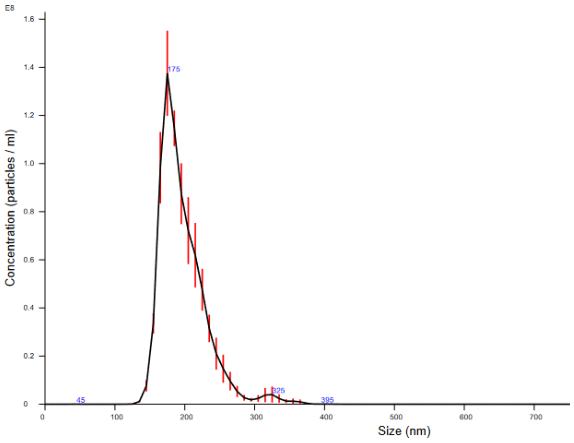


| | | | |
|---|---|---|---|
| Mean | 197.2 nm | D10 | 153.5 nm |
| Mode | 176.3 nm | D50 | 179.1 nm |
| SD | 34.0 nm | D90 | 228.5 nm |

**PLGA/BrijS20-amine-FA-NPs**

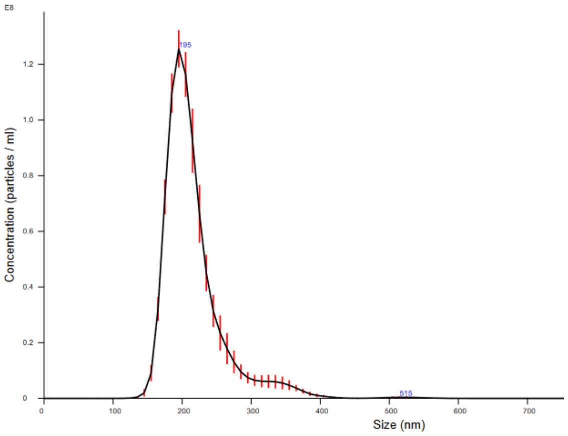


| | | | |
|---|---|---|---|
| Mean | 215.9 nm | D10 | 165.3 nm |
| Mode | 196.3 nm | D50 | 195.2 nm |
| SD | 45.3 nm | D90 | 257.8 nm |

Figure S3: Particle size distribution determined by nanoparticle tracking analysis. Average of five measurements is plotted.

## Physicochemical characterisation of BrijS0-amine-folic acid conjugate – Methods and Results

***Ultraviolet (UV) absorption spectra:***

UV-spectra of the parent compounds BrijS20 and folic acid as well as the BrijS20-amine-folic acid conjugate were recorded after dissolution in an aqueous NaOH solution (1mM) using a Hitachi U-3000 spectrophotometer. The UV-spectra of BrijS20 showed no relevant absorption between 220 nm and 700 nm. Folic acid exhibits absorption maxima at 255 nm, 285 nm, and 365 nm. The final BrijS20-amine-folic acid conjugate showed a comparable UV- absorption spectrum like folic acid confirming the presence of the targeting molecule in the final product (Figure S3).

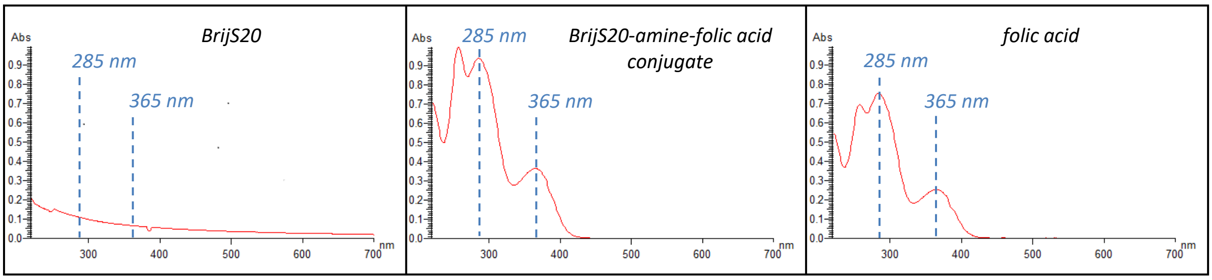


**Figure S4:** UV-absorption spectra of BrijS20, the final BrijS20-amine- folic acid conjugate and folic acid.

***Fourier-transformed infrared spectroscopy (FT-IR):***

The chemical functionalization of BrijS20 was characterised using FTIR-analysis. Infrared spectroscopic analysis in ATR mode was performed on BrijS20, folic acid as well as the final BrijS20-amine-folic acid conjugate using a Tensor 27 (Bruker Optics, Ettlingen, Germany). The sample was placed on the sample holder, pressed and scanned over a spectral range of 4000-850 $cm^{-1}$ at a spectral resolution of 4 $cm^{-1}$. FTIR spectra were analysed using OPUS 5.5 analysis software (Bruker Optics, Germany).

The final BrijS20-amine-folic acid conjugate showed signals from the parent compounds BrijS20 as well as folic acid. The characteristic stretching vibrations between 2920 $cm^{-1}$ and 2850 $cm^{-1}$ deriving from the alkyl chain of the BrijS20 moiety are clearly visible in both spectra (Figure S4). Further signals from folic acid in the fingerprint area were detected in the spectrum of the BrijS20-amine-FA conjugate. Also the additional vibrations between 3300 $cm^{-1}$ and 3100 $cm^{-1}$ from hydrogen bond interactions of the hydroxyl and amine groups in the pteroid unit of folic acid are visible in the spectrum of the final conjugate.

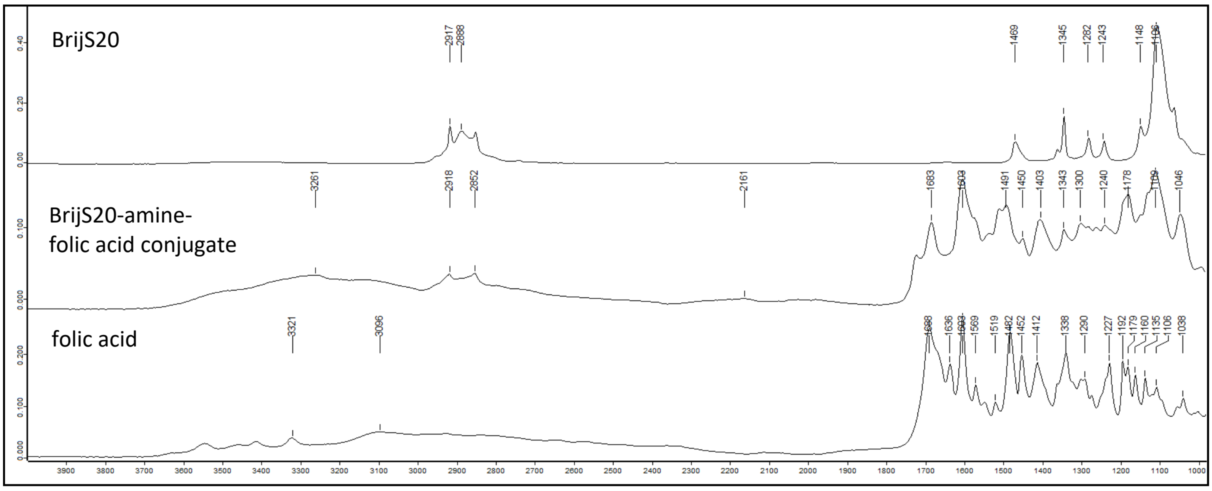


Figure S5: FT-IR spectrum of BrijS20, the final BrijS20-amine- folic acid conjugate and folic acid. Characteristic vibrations of the BrijS20 compound as well as folic acid are visible in the spectrum of the BrijS20-conjugate.

***$^{1}$H-NMR and $^{13}$C-NMR peak assignment and spectra***

NMR analysis was performed on a Bruker instrument at 400 MHz for $^{1}$H-NMR and 101 MHz for $^{13}$C-NMR spectra. $^{1}$H- and $^{13}$C-NMR spectra of the BrijS20-amine folic acid conjugate showed signals originating from the Brij-component as well as folic acid, therefore indicating the successful conjugation reaction. (Figure S5–S11) Also the formation of the intermediate products folic-acid NHS-ester and BrijS20-amine could be verified by NMR analysis.

***Mass spectrometric analysis of the BrijS20-amine folic acid conjugate***

High resolution mass spectrometric detection was performed using turbo ion source ESI X500 QTOF mass spectrometer (AB Sciex, Darmstadt, Germany). In the mass spectrum of BrijS20-amine-folic acid a mass peak from the final compound could be found at 1749 Da (Figure S12 and Table S1).

**BrijS20-amine:**

$^{1}$H NMR (400 MHz, $D_2O$): δ (ppm) 4.23 (2H, -COO-C$H_2$-), 3.53 – 3.88 (PEG-C$H_2$, m), 3.47 (2H, d, J = 7.1 Hz, $CH_3$-$(CH_2)_{15}$-$CH_2$-C$H_2$-O-), 3.35 (2H, d, *J* = 5.9 Hz, -NH-C$H_2$-), 2.87 (2H, $NH_2$-C$H_2$-), 1.58 (2H, s, -O-$CH_2$-C$H_2$-), 1.31 (30H, 15x -C$H_2$-), 0.91 (3H, t, J = 6.4 Hz, -C$H_3$).

$^{13}$C NMR (101 MHz, $D_2O$): δ (ppm) 69.72 (PEG-*C*$H_2$), 32.05 (-NH-*C*$H_2$-), 30.05 ($NH_2$-*C*$H_2$-), 29.56 (-O-$CH_2$-*C*$H_2$-), 26.23 (-O-$CH_2$-$CH_2$- *C*$H_2$), 22.71 (aliphatic *C*$H_2$), 13.94 (-*C*$H_3$).

**Folic acid NHS-ester:**

$^1$H NMR (400 MHz, DMSO-$d_6$): δ (ppm) 8.66 (1H, s, 7-C*H*), 8.05 (1H, d, *J* = 7.5 Hz, 18-N*H*), 7.64 (2H, d, *J* = 8.5 Hz, 2x 13/15-C*H*), 6.92 (1H, t, *J* = 6.0 Hz, 10-N*H*), 6.63 (2H, dd, *J* = 13.0, 8.6 Hz, 2x 12/16-C*H*), 4.49 (2H, d, *J* = 6.2 Hz, 9-C$H_2$), 4.31 (1H, q, *J* = 7.5 Hz, 19-C*H*), 2.57 (NHS-4*H*), 2.40 – 2.30 (2H, m, 22-C$H_2$), 1.97 (2H, ddt, *J* = 41.0, 13.9, 7.0 Hz, 21-C$H_2$). **residual triethylamine from synthesis: δ (ppm) 2.98, 1.13*

$^{13}$C NMR (101 MHz, DMSO-$d_6$): δ (ppm) 174.00 (20-*C*OOH), 173.77 (23-*C*ONH), 172.65 (NHS-*C*=O), 166 (17-*C*ONH), 161.08 (4-*C*=O), 153.86 (8a-*C*), 150.71 (11-NH*C*), 148.44 (6/7-*C*), 128.85 (13/15-*C*), 121.45 (14-*C*), 111.18 (12/16-*C*), 51.91 (19-*C*), 45.91 (9-*C*$H_2$), 30.68 (22-*C*$H_2$), 26.38 (21-*C*$H_2$), 25.18 (NHS-*C*$H_2$).
**residual trimethylamine (TEA) from synthesis: 45.62 (TEA-$CH_2$), 9.10 (TEA-$CH_3$)*
solvent peak (DMSO): 38-41 ppm

**BrijS20-amine-FA:**

$^1$H NMR (400 MHz, $D_2O$/NaOH): δ (ppm) 8.63 (1H, s, 7-C*H*), 7.71 (2H, dd, *J* = 8.8, 3.4 Hz, 2x 13/15-C*H*), 6.89 – 6.84 (2H, m, 2x 12/16-C*H*), 3.92 – 3.17 (PEG-C$H_2$, m), 2.30 (2H, t, *J* = 8.30 Hz, 22-C$H_2$), 2.20 – 2.09 (1H, m, 21-C$H_2$), 2.07 – 1.96 (m, 1H, 21-C$H_2$), 1.59 (2H, s, -O-$CH_2$-C$H_2$-), 1.32 (15x -C$H_2$-), 0.92 (3H, t, J = 6.6 Hz, -C$H_3$). *#residual HEPES from dialysis: δ (ppm) 3.1, 2.8, 2.6;*

$^{13}$C NMR-DEPT (101 MHz, $D_2O$): δ (ppm) 147.42 (7-CH), 129.13 (13/15-CH), 112.81 (12/16-CH), 69.64 (PEG-$CH_2$), 56.20 (19-CH), 46.09 (9-$CH_2$), 34.34 (A/B-$CH_2$), 29.99 (22-$CH_2$), 26.14 (21-$CH_2$), 23.86 (Brij-$CH_2$). *#residual HEPES from dialysis: 58.82, 58.20, 52.15, 51.44, 47.55;*

$^{13}$C NMR (101 MHz, $D_2O$): δ (ppm) 147.20 (7-*C*H), 129.18 ((13/15-*C*H), 112.88 (12/16-CH), 69.71 (PEG-*C*$H_2$), 46.16 (9-$CH_2$), 34.38 (A/B-*C*$H_2$), 30.01 (22-*C*$H_2$), 26.23 (21-*C*$H_2$), 22.72 (Brij-*C*$H_2$), 13.97 (-*C*$H_3$).
*#residual HEPES from dialysis: 58.88, 58.27, 52.20, 51.49, 47.61*

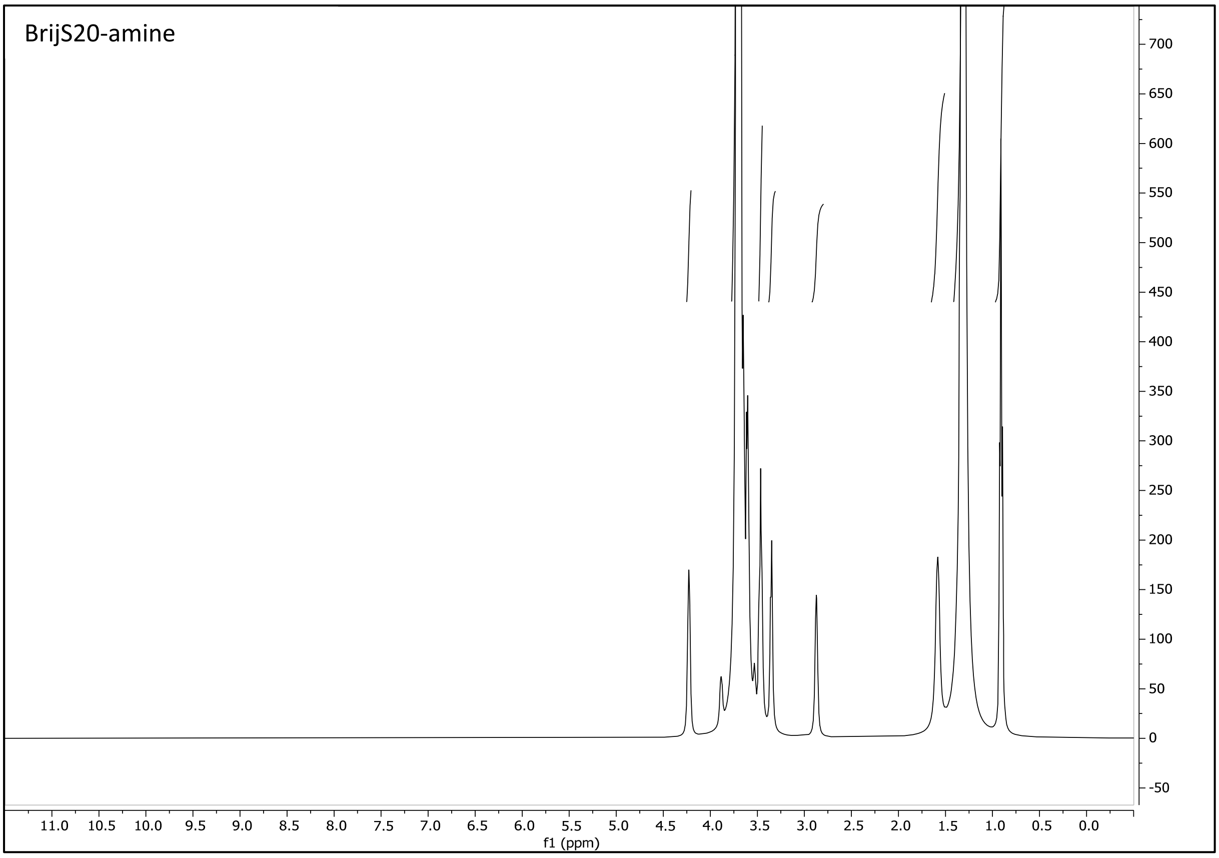


*Figure S6: $^{1}$H-NMR of BrijS20-amine in $D_2O$.*

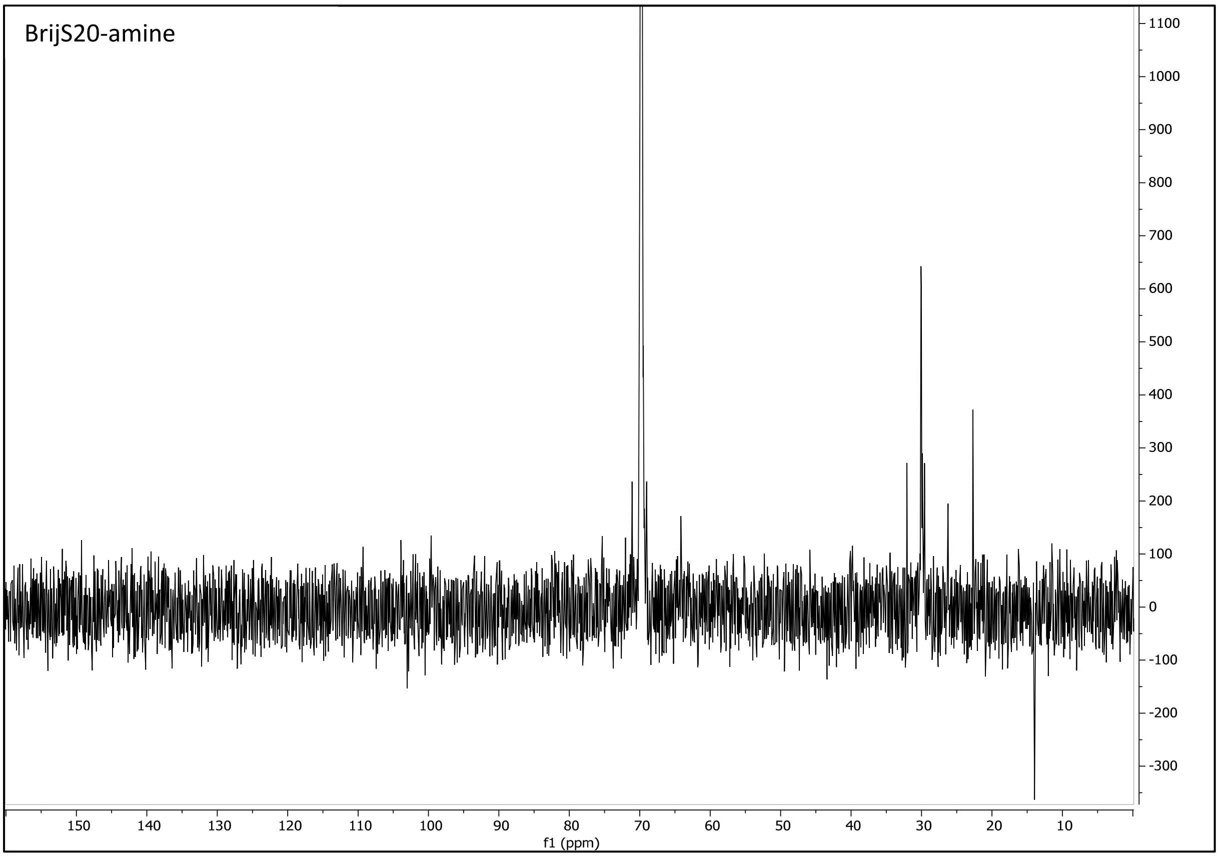


*Figure S7: $^{13}$C-NMR of BrijS20-amine in $D_2O$.*

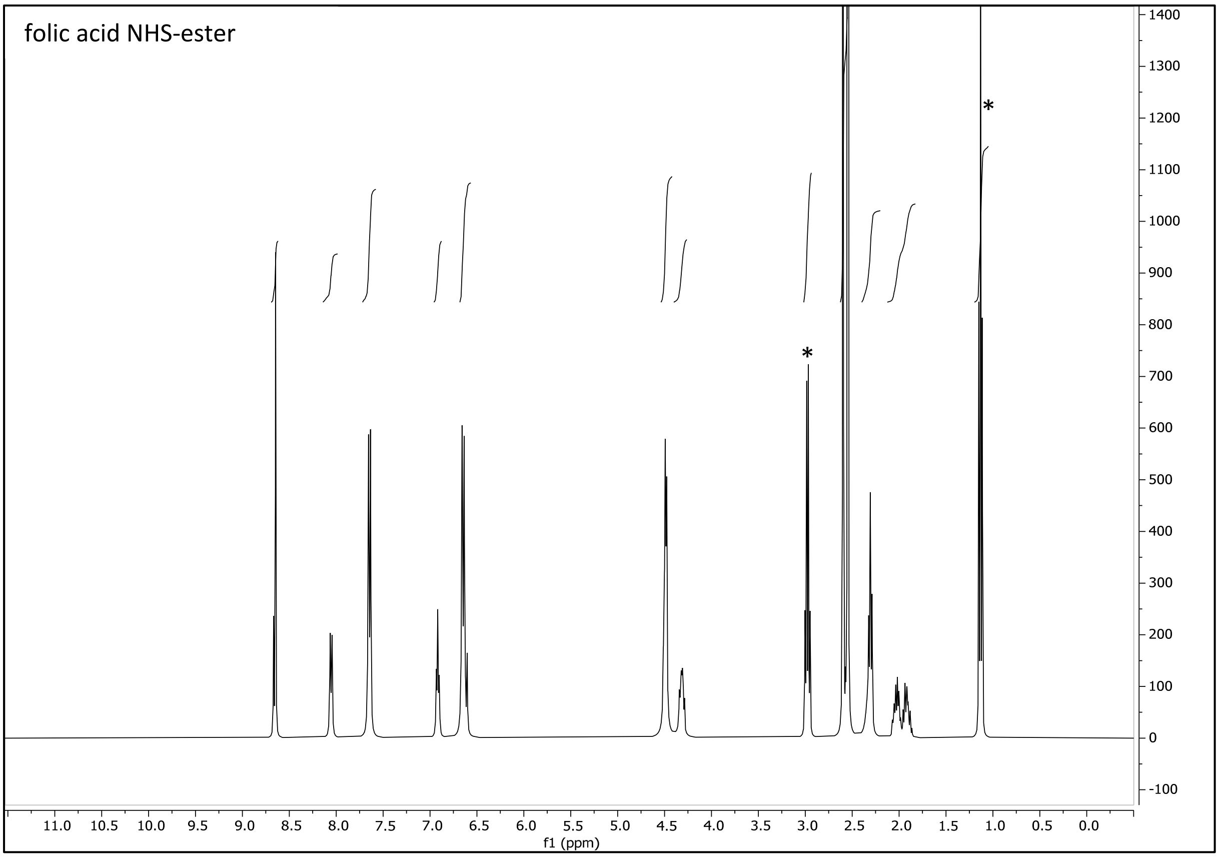

*Figure S8: $^{1}$H-NMR of folic acid NHS-ester in DMSO-d$_6$.* **residual triethylamine from synthesis*

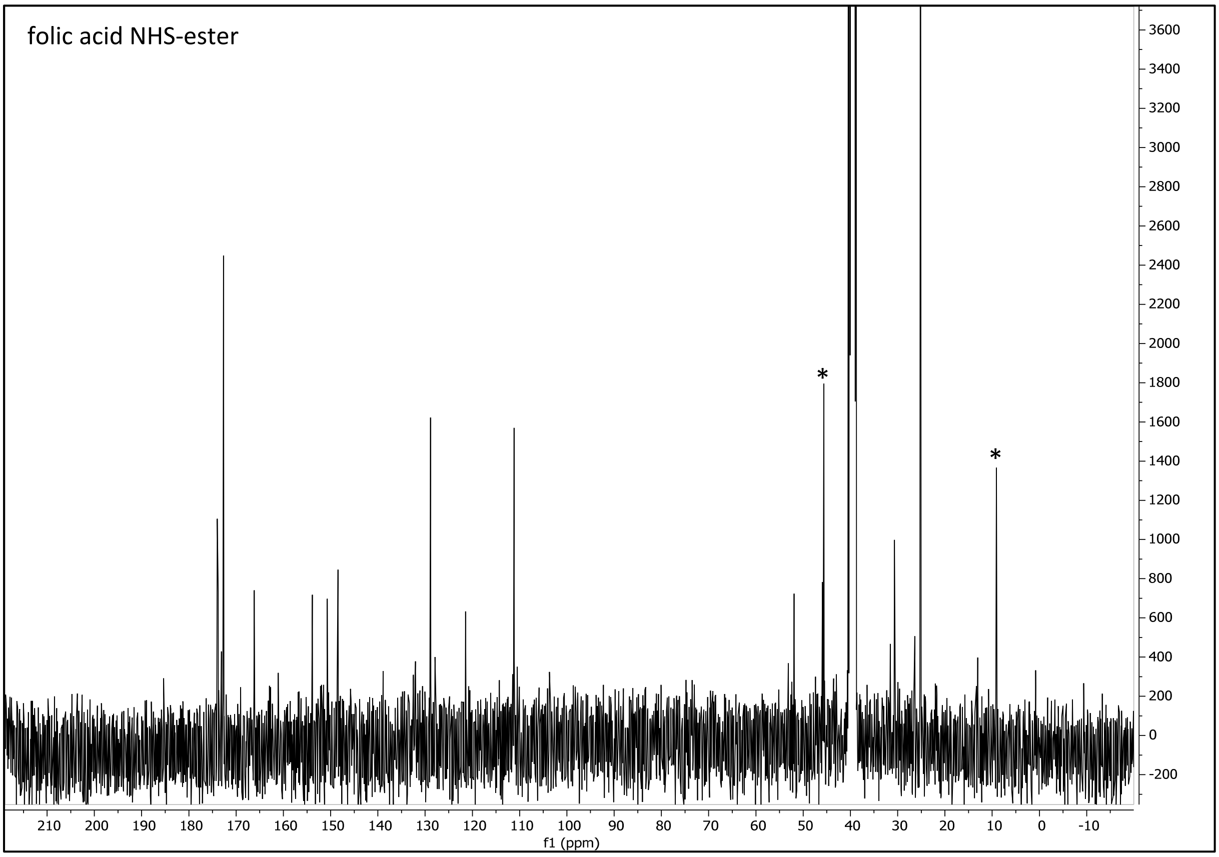

*Figure S9: $^{13}$C-NMR of folic acid NHS-ester in DMSO-d$_6$.* **residual triethylamine from synthesis; DMSO-d$_6$ solvent peak: 38-41 ppm*

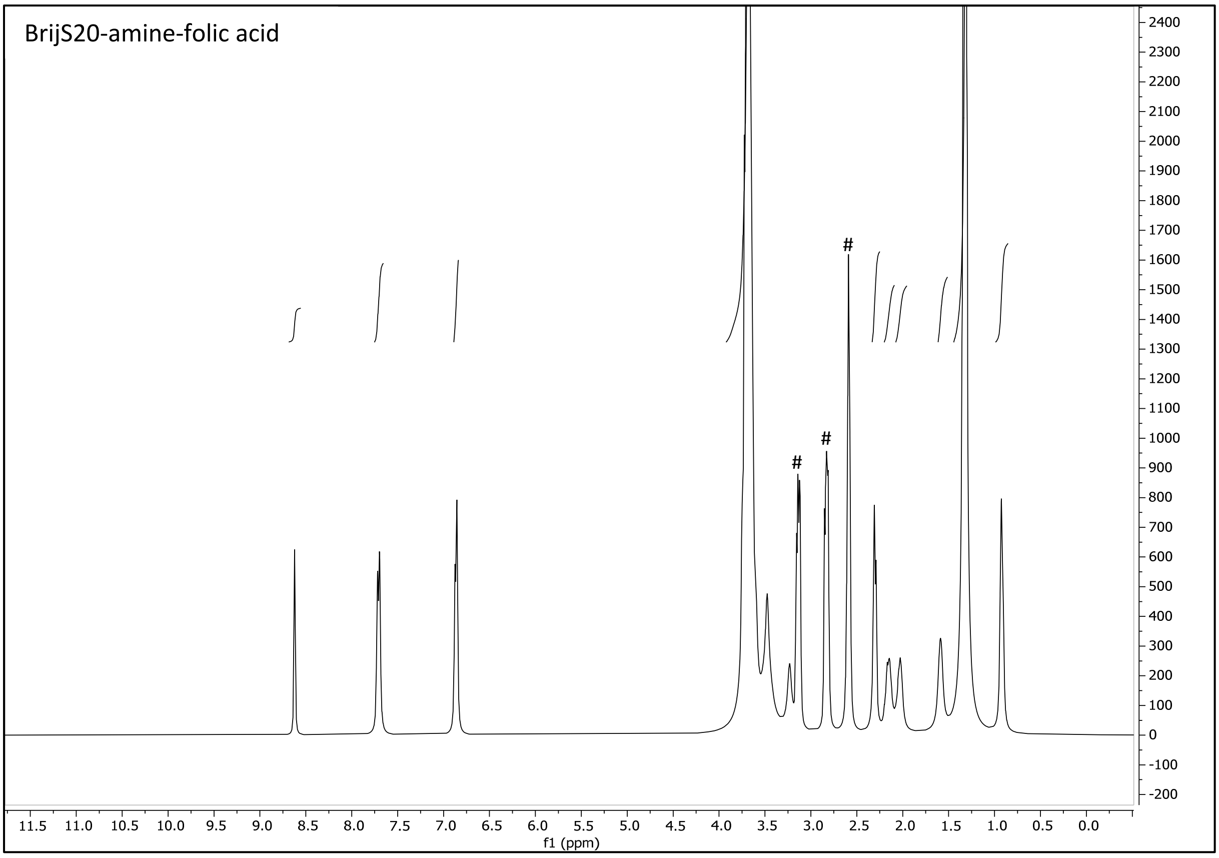


*Figure S10: $^{1}$H-NMR of BrijS20-amine folic acid in $D_2O$/NaOH.* *#residual HEPES from dialysis*

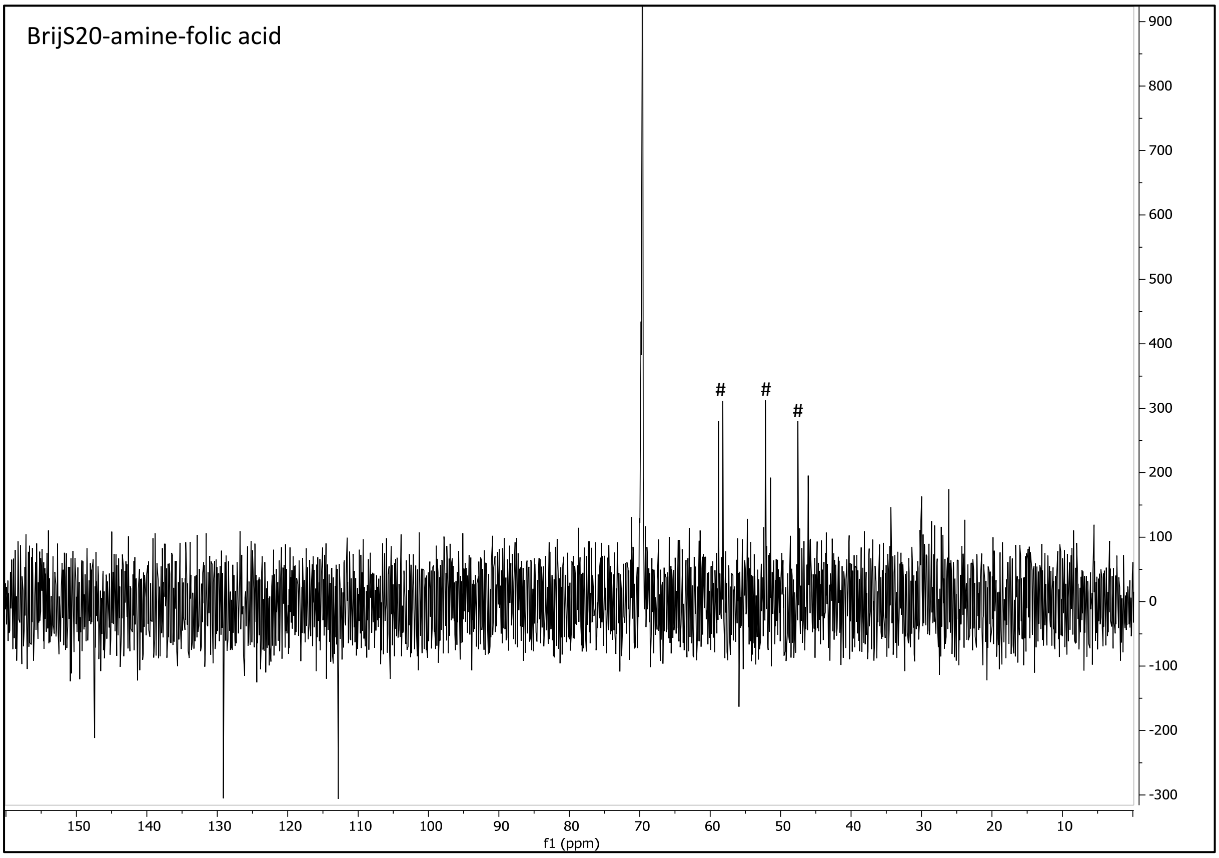


*Figure S11: $^{13}$C-NMR-DEPT of BrijS20-amine folic acid in $D_2O$/NaOH.* *#residual HEPES from dialysis*

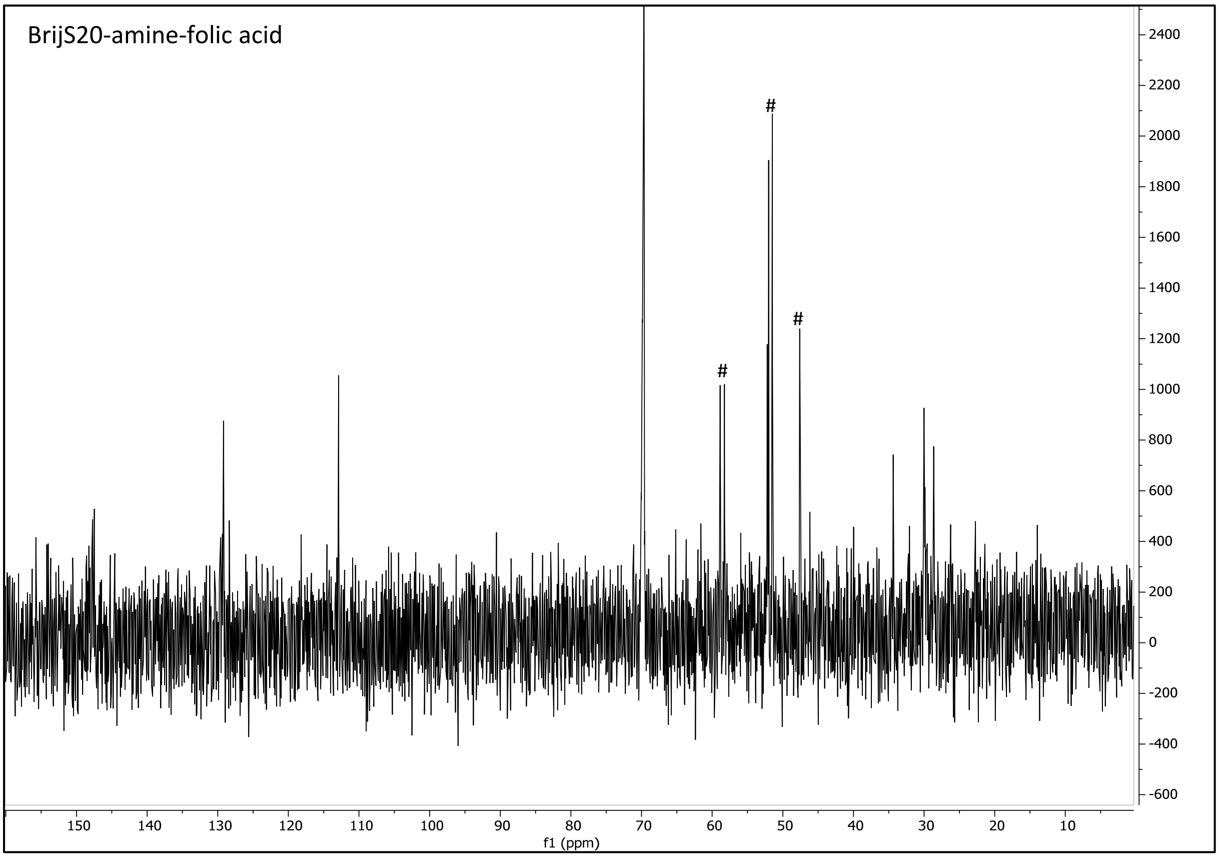


*Figure S12: $^{13}$C-NMR of BrijS20-amine folic acid in $D_2O$/NaOH.* *#residual HEPES from dialysis*

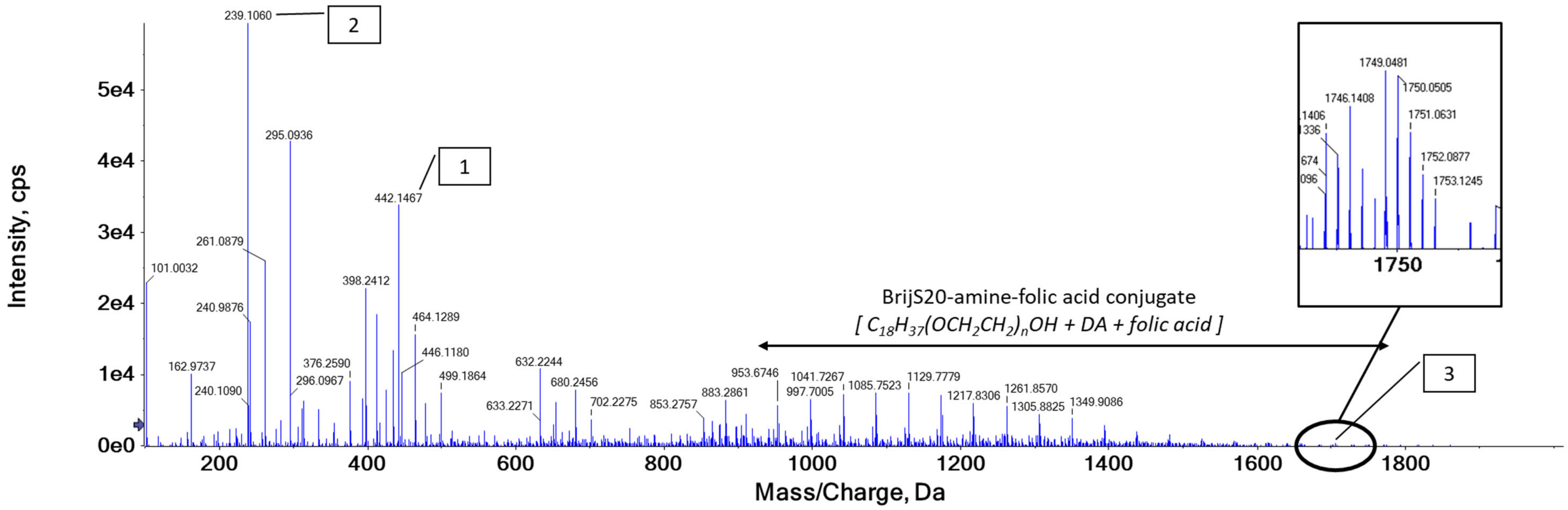


*Figure S13: mass spectrum of BrijS20-amine-folic acid conjugate. The m/z region of the final product [3] is enlarged.*

*Table S1: exact mass and [m +H] of characteristic peaks in the mass spectrum of the BrijS20-amine-folic acid conjugate. Next to the product ion peak, an ion peak series reflecting the different degrees of pegylation of the parent Brij component can be identified.*

| # | sum formula | [M+H] | exact mass | name |
|---|---|---|---|---|
| 1 | $C_{19}H_{19}N_7O_6$ | 442.1467 Da | 441.1396 Da | Folic Acid |
| 2 | $C_8H_{18}N_2O_4S$ | 239.1065 Da | 238.0987 Da | HEPES |
| 3 | $C_{84}H_{149}N_9O_{29}$ | 1749.0481 Da | 1748.0461 Da | BrijS20-amine-folic acid conjugate |

Suggested structures:

**BrijS20-amine**

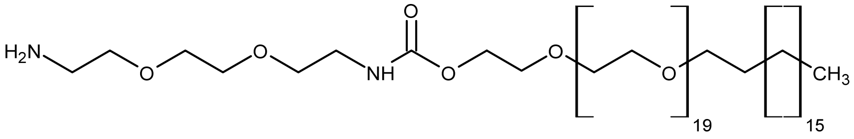

**Folic acid NHS-ester:**

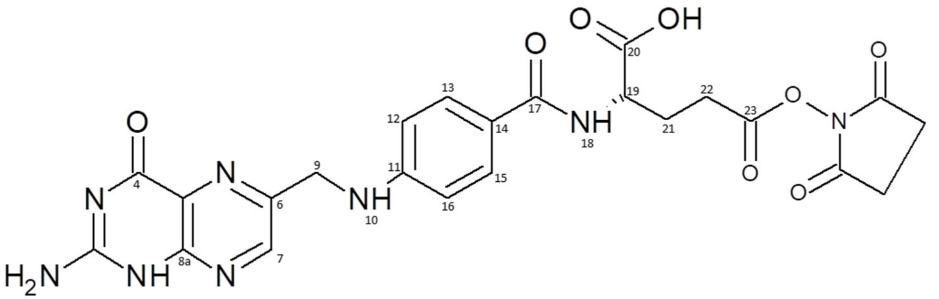

**BrijS20-amine-FA:**

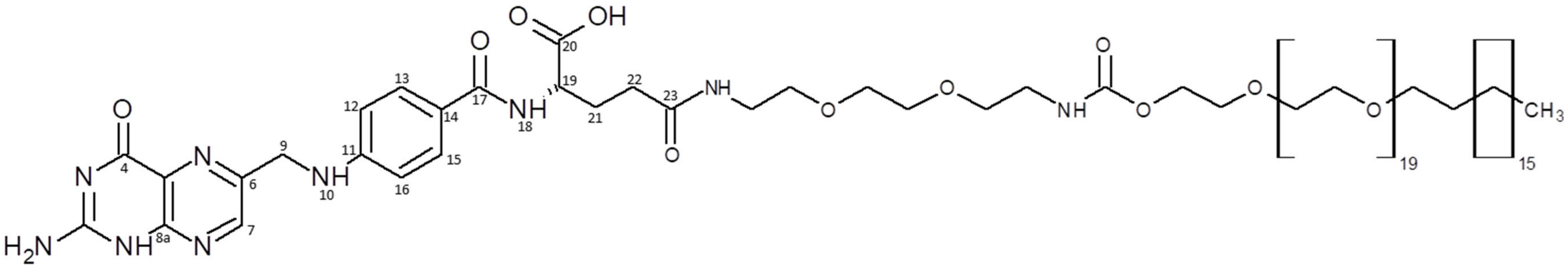

Supplement: Supplementary file 1 [file pharmaceutics-15-01403-s001.zip › pharmaceutics-2332023-supplementary.pdf]
